# Two novel saprobic *Distoseptispora* species from southern China (Distoseptisporales, Distoseptisporaceae)

**DOI:** 10.3897/mycokeys.132.188292

**Published:** 2026-05-13

**Authors:** Xing-Guo Tian, Jia-Jun Han, Yong-Zhong Lu, Samantha C. Karunarathna, Saowaluck Tibpromma, Dan-Feng Bao

**Affiliations:** 1 School of Food and Pharmaceutical Engineering, Guizhou Institute of Technology, Guiyang, 550025, China Guizhou Key Laboratory of Agricultural Microbiology, Guizhou Academy of Agricultural Sciences Guiyang China; 2 Guizhou Key Laboratory of Agricultural Microbiology, Guizhou Academy of Agricultural Sciences, Guiyang, 550009, China College of Biology and Food Engineering, Qujing Normal University Qujing China; 3 Department of Entomology and Plant Pathology, Faculty of Agriculture, Chiang Mai University, Chiang Mai, 50200, Thailand Faculty of Agriculture, Chiang Mai University Chiang Mai Thailand; 4 Center for Yunnan Plateau Biological Resources Protection and Utilization & Yunnan International Joint Laboratory of Fungal Sustainable Utilization in South and Southeast Asia, College of Biology and Food Engineering, Qujing Normal University, Qujing, 655099, China School of Food and Pharmaceutical Engineering, Guizhou Institute of Technology Guiyang China

**Keywords:** 2 new species, Distoseptisporaceae, morphology, phylogeny, taxonomy

## Abstract

*Distoseptispora* comprises ecologically significant species that are predominantly saprobic, inhabiting freshwater and terrestrial ecosystems, where they contribute to nutrient cycling and organic matter decomposition. Recent taxonomic studies have revealed several new *Distoseptispora* species, highlighting its high diversity. In this study, we describe two new species, *Distoseptispora guanxiensis* and *D. longiconidiophora*, isolated from decaying wood in southern China, thereby further expanding the known species richness of the genus. Based on multi-locus phylogenetic analyses integrated with the internal transcribed spacer (ITS), the large ribosomal subunit of the nuclear ribosomal DNA (LSU), the RNA polymerase II second largest subunit (*rpb*2), and the translation elongation factor 1-alpha (*tef*1-α), our isolates of *D. guanxiensis* formed a well-supported lineage sister to *D. rayongensis*, while *D. longiconidiophora* formed a basal position relative to the clade comprising *D. cangshanensis*, *D. cylindricospora*, *D. hongheensis* and *D. pulchra*. Morphological characteristics of the new species are compared with their phylogenetically close relatives, and detailed descriptions, illustrations, and phylogenetic data are provided. This study highlights the high diversity and distribution of *Distoseptispora* species, and emphasizes the importance of integrating morphological and molecular data for accurate species identification.

## Introduction

*Distoseptispora*, the sole genus in Distoseptisporaceae (Distoseptisporales), was introduced by Su et al. (2016) with *D. fluminicola* as the type species. Since its establishment, the genus has expanded rapidly, with 124 species described based on morphological characteristics and phylogenetic analysis (Index Fungorum 2026). Species in this genus are primarily saprobes inhabiting freshwater and terrestrial habitats, where they play important roles in nutrient cycling in forest ecosystems (Hyde et al. 2016; Tibpromma et al. 2018; Phookamsak et al. 2019; Phukhamsakda et al. 2020; Shen et al. 2021, 2024; Zhang et al. 2022; Liao et al. 2025; Liu et al. 2025).

*Distoseptispora* is predominantly known from its asexual morphs, characterized by macronematous, unbranched, septate conidiophores; monoblastic or polyblastic, integrated, terminal, determinate or percurrently extending conidiogenous cells; and acrogenous, solitary, distoseptate or euseptate conidia (Su et al. 2016). Intraspecific variation in septation and in the size of conidia and conidiophores is common, making species identification based solely on morphology challenging. For example, Yang et al. (2018) reported that *D. multiseptata* exhibited conidia of up to 380 μm in some collections and up to 700 μm in others. This morphological plasticity underscores the necessity of multi-locus phylogenetic analyses (e.g., ITS, LSU, *rpb*2, and *tef*1-α) to accurately delineate species. Phylogenetic studies revealed three major clades within the genus, yet morphological traits do not consistently correspond with these clades, highlighting the need for refined systematic criteria (Zhang et al. 2022). The sexual morph of *Distoseptispora* is poorly documented, with only four species, *D. euseptata*, *D. hyalina*, *D. licualae*, and *D. suoluoensis* reported in their sexual stage (Yang et al. 2018; Li et al. 2021; Konta et al. 2023). These are characterized by immersed to semi-immersed, subglobose to ellipsoidal, dark-brown perithecia with short necks; a relatively thick peridia; sparse, persistent, hyaline paraphyses; cylindrical asci with non-amyloid apical annuli; and hyaline, filiform, septate ascospores with mucilaginous sheaths (Yang et al. 2018).

Species within this genus are highly diverse in China and Thailand, particularly in southern China, including Guizhou (Liao et al. 2025), Jiangxi provinces (Hu et al. 2023) and Yunnan (Shen et al. 2021, 2024), which are emerging as biodiversity hotspots for *Distoseptispora* species. Despite these advances, the taxonomy of *Distoseptispora* remains dynamic, with ongoing discoveries of novel species through molecular phylogenetics and intensified field surveys. For instance, three new species, *D. meilingensis*, *D. yongxiuensis*, and *D. yunjushanensis*, were recently described from submerged bamboo culms in Jiangxi Province (Zhai et al. 2022), underscoring the ecological diversity of freshwater habitats. Likewise, studies in Yunnan have revealed taxa such as *D. mengsongensis* and *D. nabanheensis* (Liu et al. 2023), highlighting the province’s role as a reservoir of underexplored fungal biodiversity.

In this study, two new *Distoseptispora* species were collected and isolated from southern China. We used integrative taxonomy, combining morphological observations with phylogenetic analyses of concatenated nuclear ribosomal DNA (ITS-LSU) and protein-coding genes (*rpb*2-*tef*1-α). Our objectives are to provide morphological and molecular evidence for these novel species and to contribute to a broader understanding of fungal biodiversity and biogeography within the genus.

## Materials and methods

### Isolation and morphology

Specimens of decaying wood were collected from Fangchenggang City, Guangxi Zhuang Autonomous Region, and Xishuangbanna City, Yunnan Province, China during 2021–2024. Collection details were recorded following Rathnayaka et al. (2025), and the samples were placed in plastic bags and taken to the laboratory. Specimen observations and morphological studies were conducted according to the protocols described by Luo et al. (2018) and Tian et al. (2026).

Single spore isolations were performed following the method described by Senanayake et al. (2020). Germinating conidia were transferred aseptically to potato dextrose agar (PDA) plates and grown at 16 °C under daylight conditions. Colony colour and other characteristics were observed and measured after one week and again after three weeks.

The specimens were deposited in the Guizhou Academy of Agriculture Sciences Culture Collection (GZCC), and the corresponding herbarium specimens are preserved at the Guizhou Academy of Agriculture Sciences (Herb. GZAAS). Facesoffungi numbers (FoF) were assigned following Jayasiri et al. (2015), and Index Fungorum numbers (IF) were obtained from http://www.indexfungorum.org/names/names.asp.

### DNA extraction, PCR amplification, and sequencing

Mycelia were scraped from the surface of colonies grown on PDA plates at 25 °C for 4 weeks, transferred into 1.5 mL centrifuge tubes, and ground in liquid nitrogen. Genomic DNA was extracted from the ground mycelia using the EZgene™ Fungal gDNA Kit (GD2416; Biomiga, USA) following the manufacturer’s instructions. The loci of ITS, LSU, *tef*1-α, and *rpb*2, were amplified using the primer pairs ITS5/ITS4 (White et al. 1990), LR0R/LR7 (Vilgalys and Hester 1990), 983F/2218R (Rehner 2001), and fRPB2-5F/fRPB2-7cR (Liu et al. 1999), respectively. The PCR mixture (25 μL total volume) contained 12.5 μl of 2× Power Taq PCR MasterMix (a premix comprising 0.1 Units/μL Taq DNA Polymerase, 500 μM each dNTP, 20 mM Tris-HCl pH 8.3, 100 mM KCl, 3 mM MgCl_2_), 1 μl of each primer (10 μm), 1 μl genomic DNA, and 9.5 μl deionized water. Polymerase Chain Reaction amplification was performed under the following conditions: initial denaturation at 94 °C for 3 min; 35 cycles of denaturation at 94 °C for 30 s, annealing at 56 °C for 50 s, and elongation at 72 °C for 1 min; followed by a final extension at 72 °C for 10 min, and then held at 4 °C (Tian et al. 2025). The PCR amplificons were confirmed on 1% agarose gels stained with ethidium bromide. Purification and sequencing of PCR products were carried out at Shanghai Sangon Biological Engineering Technology and Services Co., Ltd (Shanghai, P.R. China).

### Phylogenetic analyses

Sequences were assembled using BioEdit, and BLAST searches were conducted to identify the closest matches to taxa in Distoseptisporaceae, based on recently published data (Liao et al. 2025). All consensus sequences and reference sequences were aligned using MAFFT v.7 (http://mafft.cbrc.jp/alignment/server/index.html; Katoh et al. 2019). Ambiguously aligned regions and uninformative positions were removed using TrimAl v1.2 with the “gt = 0.3” option (Capella-Gutiérrez et al. 2009). Sequences for each locus were concatenated using SequenceMatrix 1.7.8 (Vaidya et al. 2011), and phylogenetic analyses were performed using both Maximum Likelihood (ML) and Bayesian analysis (Table 1).

**Table 1.** Taxa used in the phylogenetic analyses and their corresponding GenBank accession numbers.

Taxa	Strain Number	GenBank Accession numbers			
		LSU	ITS	*tef*1-α	*rpb*2
*Acrodictys bambusicola*	CGMCC 3.18641 T	KX033564	KU999973	—	—
*Acrodictys elaeidicola*	CGMCC 3.18642	KX033569	KU999978	—	—
*Aquapteridospora aquatica*	MFLUCC 17-2371 T	MW287767	MW286493	—	—
*Aquapteridospora fusiformis*	MFLUCC 18-1606 T	MK849798	MK828652	MN194056	—
*Aquapteridospora lignicola*	MFLUCC 15-0377 T	KU221018	MZ868774	MZ892980	MZ892986
*Bullimyces aurisporus*	AF316-1b T	JF775590	—	—	—
*Bullimyces communis*	AF281-5	JF775587	—	—	—
*Cancellidium applanatum*	CBS 337.76 T	MH872755	MH860985	—	—
*Cancellidium cinereum*	MFLUCC 18-0424 T	MT370363	MT370353	MT370488	MT370486
*Distoseptispora adscendens*	HKUCC 10820	DQ408561	—	—	DQ435092
*Distoseptispora amniculi*	MFLUCC 17-2129 T	MZ868761	MZ868770	—	MZ892982
*Distoseptispora appendiculata*	MFLUCC 18-0259 T	MN163023	MN163009	MN174866	—
*Distoseptispora aqualignicola*	KUNCC 21-10729 T	ON400845	OK341186	OP413480	OP413474
*Distoseptispora aquamyces*	KUNCC 21-10732 T	OK341199	OK341187	OP413482	OP413476
*Distoseptispora aquatica*	MFLUCC 18-0646	MK849793	MK828648	MN194052	—
*Distoseptispora aquatica*	MFLUCC 15-0374T	KU376268	MF077552	—	—
*Distoseptispora aquisubtropica*	GZCC 22-0075 T	ON527941	ON527933	ON533677	ON533685
*Distoseptispora arecacearum*	MFLUCC 23-0212	OR510860	OR354399	OR481045	OR481048
*Distoseptispora arecacearum*	MFLUCC_23_0211T	OR510857	OR234707	OR250439	OR250442
*Distoseptispora atroviridis*	GZCC 20-0511 T	MZ868763	MZ868772	MZ892978	MZ892984
*Distoseptispora atroviridis*	GZCC 19-0531	MZ227223	MW133915	MZ206155	—
*Distoseptispora bambusae*	MFLUCC 20-0091 T	NG_074430	NR_170068	MT232880	MT232881
*Distoseptispora bambusicola*	GZCC 21-0667 T	MZ474872	MZ474873	OM272845	—
*Distoseptispora bangkokensis*	MFLUCC 18-0262 T	MZ518206	MZ518205	OK067246	—
*Distoseptispora bawanglingensis*	SAUCC WZS13‐1T	PQ804721	PQ799295	PQ849363	PQ849357
*Distoseptispora cangshanensis*	MFLUCC 16-0970 T	MG979761	MG979754	MG988419	—
*Distoseptispora caricis*	CPC 36498 T	MN567632	MN562124	—	MN556805
*Distoseptispora changjiangensis*	SAUCCWZS14‐1T	PQ804723	PQ799297	PQ849366	PQ849359
*Distoseptispora chengduensis*	CGMCC 3.27439 T	PQ067744	PQ067913	PQ278565	—
*Distoseptispora chiangraiensis*	MFLU 21-0105 T	MZ890139	MZ890145	MZ892970	—
*Distoseptispora chinensis*	GZCC 21-0665 T	MZ474867	MZ474871	MZ501609	—
*Distoseptispora chishuiensis*	GZCC 23-0729 T	PP584767	PP584670	PP663310	—
*Distoseptispora clematidis*	MFLUCC 17-2145 T	MT214617	MT310661	—	MT394721
*Distoseptispora combreticola*	CGMCC 3.27721T	PQ184737	PQ189782	—	—
*Distoseptispora crassispora*	KUMCC 21-10726 T	OK341196	OK310698	OP413479	OP413473
*Distoseptispora curvularia*	KUMCC 21-10725 T	OK341195	OK310697	OP413478	OP413472
*Distoseptispora cylindricospora*	DLUCC 1906 T	OK513523	OK491122	OK524220	—
*Distoseptispora daanyuanensis*	SAUCC12326‐1T	PV670405	PV670056	PV708057	—
*Distoseptispora davidalangii*	UESTCC 24.0236T	PQ184736	PQ189781	PQ346519	PQ380000
*Distoseptispora davidii*	GZCC 24-0091T	PV856177	PV820360	—	—
*Distoseptispora dehongensis*	KUMCC 18-0090 T	MK079662	MK085061	MK087659	—
*Distoseptispora dinghuensis*	ZHKUCC 23-0958T	PQ037956	PQ037957	PQ035181	PQ035180
*Distoseptispora dipterocarpi*	MFLUCC 22-0104 T	OP600052	OP600053	—	OP595140
*Distoseptispora dujuanhuensis*	KUNCC 23-13772T	PV536297	PQ845849	PX238245	PX233767
*Distoseptispora effusa*	GZCC 19-0532 T	MZ227224	MW133916	MZ206156	—
*Distoseptispora eleiodoxae*	MFLUCC 23-0214	OR510859	OR354398	OR481044	OR481047
*Distoseptispora eleiodoxae*	MFLUCC_23-0213T	OR510856	OR234706	OR250438	OR250441
*Distoseptispora euseptata*	MFLUCC 20-0154 T	MW081544	MW081539	—	MW151860
*Distoseptispora euseptata*	DLUCC S2024	MW081545	MW081540	MW084994	MW084996
*Distoseptispora fasciculata*	KUMCC 19-0081 T	MW287775	MW286501	MW396656	—
*Distoseptispora fluminicola*	DLUCC 0391	MG979762	MG979755	MG988420	—
*Distoseptispora fluminicola*	DLUCC 0999	MG979763	MG979756	MG988421	—
*Distoseptispora fluminicola*	MFLUCC 15-0417T	KU376270	NR_154041	—	—
*Distoseptispora fujianensis*	HJAUP C2509 T	PQ211103	PQ211095	PQ303682	PQ303679
*Distoseptispora fusiformis*	GZCC 20-0512 T	MZ868764	MZ868773	MZ892979	MZ892985
*Distoseptispora ganzhouensis*	HJAUP C1090 T	PQ211108	PQ211100	PQ303687	—
*Distoseptispora gasaensis*	HJAUP C2034 T	OQ942891	OQ942896	OQ944455	—
*Distoseptispora gelatinosa*	GUCC 24-0149 T	PQ570872	PQ570855	—	—
*Distoseptispora guanshanensis*	HJAUP C1063 T	OQ942898	OQ942894	OQ944452	OQ944458
***Distoseptispora guanxiensis***	**GZCC 26-0130 T**	**PX984884**	**PX889843**	**—**	**—**
***Distoseptispora guanxiensis***	**GZCC 26-0131**	**PX984885**	**PX984883**	**PZ013918**	**PZ013919**
*Distoseptispora guizhouensis*	GZCC 21-0666 T	MZ474869	MZ474868	MZ501610	MZ501611
*Distoseptispora guttulata*	MFLU 17-0852 T	MF077554	MF077543	MF135651	—
*Distoseptispora hainanensis*	GZCC 22-2047 T	OR438894	OR427328	OR449122	OR449119
*Distoseptispora heptapleuricola*	CGMCC 3.27740T	PQ184738	PQ189783	PQ346520	PQ380001
*Distoseptispora hongheensis*	KUNCC 23-14299T	PV536299	PQ845851	PX238247	PX233769
*Distoseptispora hyalina*	MFLUCC 17-2128 T	MZ868760	MZ868769	MZ892976	MZ892981
*Distoseptispora hydei*	MFLUCC 20-0481 T	MT742830	MT734661	—	MT767128
*Distoseptispora jianfenglingensis*	SAUCCWZS65‐3T	PQ804725	PQ799299	PQ849367	PQ849361
*Distoseptispora jingdongensis*	KUNCC 23-13382T	PX245753	PQ845852	PX238248	PX233770
*Distoseptispora jinghongensis*	HJAUP C2120 T	OQ942893	OQ942897	OQ944456	—
*Distoseptispora keviniana*	GZCC 24-0289T	—	PV820358	—	—
*Distoseptispora keviniligustrina*	CGMCC 3.27722T	PQ184727	PQ191052	PQ346501	PQ379955
*Distoseptispora lancangjiangensis*	DLUCC 1864 T	MW879522	MW723055	—	—
*Distoseptispora lanceolatispora*	GZCC 22-2045 T	OR438895	OR427329	OR449123	OR449120
*Distoseptispora leonensis*	HKUCC 10822	DQ408566	—	—	DQ435089
*Distoseptispora licualae*	MFLUCC 14-1163A T	ON650675	ON650686	ON734007	—
*Distoseptispora licualae*	MFLUCC 14-1163B T	ON650676	ON650687	ON734008	—
*Distoseptispora licualae*	MFLUCC 14-1163C T	ON650677	ON650688	—	—
*Distoseptispora lignicola*	MFLUCC 18-0198 T	MK849797	MK828651	—	—
*Distoseptispora lignicola*	GZCC 19-0529	MZ227219	MW133911	MZ206152	—
*Distoseptispora liupanshuiensis*	GZCC 23-0730 T	PP584766	PP584669	PP663309	—
*Distoseptispora longispora*	HFJAU 0705 T	MH555357	MH555359	—	—
*Distoseptispora longissima*	GMBC5339T	PV932987	PV932968	PX392330	PX373356
*Distoseptispora longnanensis*	HJAUP C1040 T	OQ942886	OQ942887	OQ944451	—
***Distoseptispora longiconidiophora***	**GZCC 25-0678**	**PX889839**	**PX860094**	**—**	**—**
***Distoseptispora longiconidiophora***	**GZCC 25-0679 T**	**PX889838**	**PX860093**	**PX890982**	**PX890981**
*Distoseptispora martinii*	CGMCC 3.18651 T	KX033566	KU999975	—	—
*Distoseptispora meilingensis*	JAUCC 4727 T	OK562396	OK562390	OK562408	—
*Distoseptispora meilingensis*	JAUCC 4728 T	OK562397	OK562391	OK562409	—
*Distoseptispora menghaiensis*	HJAUP C2045 T	OQ942900	OQ942890	—	—
*Distoseptispora menglunensis*	HJAUP C2170 T	OQ942888	OQ942899	OQ944457	OQ944461
*Distoseptispora mengsongensis*	HJAUP C2126 T	OP787874	OP787876	OP961937	—
*Distoseptispora monospora*	KAS 145690T	PQ898699	PQ898696	PV001669	PV001672
*Distoseptispora motuoensis*	KUNCC24-17628 T	PP621731	PP600327	PP639546	—
*Distoseptispora muchuanensis*	CGMCC 3.27444 T	NG_244352	NR_199255	PQ278571	—
*Distoseptispora multiseptata*	MFLUCC 15-0609 T	KX710140	KX710145	MF135659	—
*Distoseptispora multiseptata*	MFLU 17-0856	MF077555	MF077544	MF135652	MF135644
*Distoseptispora nabanheensis*	HJAUP C2003 T	OP787877	OP787873	OP961935	—
*Distoseptispora nanchangensis*	HJAUP C1074 T	OQ942895	OQ942889	OQ944454	OQ944460
*Distoseptispora nanpingensis*	HJAUP C2517 T	PQ211104	PQ211096	—	PQ303678
*Distoseptispora narathiwatensis*	MFLUCC 23-0216	OR510861	OR354400	OR481046	OR481049
*Distoseptispora narathiwatensis*	MFLUCC 23-0215T	OR510858	OR234708	OR250440	OR250443
*Distoseptispora neorostrata*	MFLUCC 18-0376 T	MN163017	MN163008	—	—
*Distoseptispora nonrostrata*	KUNCC 21-10730 T	OK341198	OK310699	OP413481	OP413475
*Distoseptispora obclavata*	MFLUCC 18-0329 T	MN163010	MN163012	—	—
*Distoseptispora obpyriformis*	MFLUCC 17-1694 T	MG979764	—	MG988422	MG988415
*Distoseptispora obpyriformis*	DLUCC 0867	MG979765	MG979757	MG988423	MG988416
*Distoseptispora olivaceoviridis*	MFLUCC 24-0299T	PQ569325	PQ568144	—	—
*Distoseptispora pachyconidia*	KUMCC 21-10724 T	OK341194	OK310696	OP413477	OP413471
*Distoseptispora palmarum*	MFLUCC 18-1446 T	MK079663	MK085062	MK087660	MK087670
*Distoseptispora phangngaensis*	MFLUCC 16-0857 T	MF077556	MF077545	MF135653	—
*Distoseptispora phragmiticola*	GUCC 22-0201 T	OP749880	OP749887	OP749891	OP752699
*Distoseptispora phragmiticola*	GUCC 22-0202 T	OP749881	OP749888	OP749892	OP752700
*Distoseptispora polyblasta*	GZCC 25-0526T	PV856176	PV820359	—	—
*Distoseptispora pseudoaquisubtropica*	GZCC 24-0061T	PV856178	PV820361	—	—
*Distoseptispora pulchra*	KUNCC 23-17029T	PQ576433	PQ469943	PQ587327	PQ587326
*Distoseptispora quinqueseptata*	GMBC 5341T	PV932985	PV932966	PX392328	PX373358
*Distoseptispora rayongensis*	MFLUCC 18-0415 T	MH457137	MH457172	MH463253	MH463255
*Distoseptispora rayongensis*	MFLUCC 18-0417	MH457138	MH457173	MH463254	MH463256
*Distoseptispora rostrata*	MFLUCC 16-0969 T	MG979766	MG979758	MG988424	MG988417
*Distoseptispora rostrata*	DLUCC 0885	MG979767	MG979759	MG988425	—
*Distoseptispora saprophytica*	MFLUCC 18-1238 T	MW287780	MW286506	MW396651	MW504069
*Distoseptispora septata*	GZCC 22-0078 T	ON527947	ON527939	ON533683	ON533690
*Distoseptispora sichuanensis*	KUNCC 23-15519	PP584769	PP584672	PP663312	—
*Distoseptispora sichuanensis*	KUNCC_23_15518T	PP584768	PP584671	PP663311	
*Distoseptispora sinensis*	HJAUP C2044 T	OP787875	OP787878	OP961936	—
*Distoseptispora solitaria*	GZCC 24-0038T	PV856179	PV820362	—	—
*Distoseptispora songkhlaensis*	MFLUCC 18-1234T	MW287755	MW286482	MW396642	—
*Distoseptispora suae*	CGMCC 3.24262 T	OQ732679	OQ874968	OR367670	OQ870341
*Distoseptispora submersa*	MFLUCC_16_0946_T	MG979768	NR_157553	MG988426	MG988418
*Distoseptispora subtropica*	HJAUP C2528 T	PQ211107	PQ211099	PQ303684	PQ303677
*Distoseptispora suoluoensis*	MFLUCC 17-0224 T	MF077557	MF077546	MF135654	—
*Distoseptispora suoluoensis*	MFLUCC 17-0854	MF077558	MF077547	—	MZ945510
*Distoseptispora tectonae*	MFLUCC 12-0291 T	KX751713	KX751711	KX751710	KX751708
*Distoseptispora tectonae*	MFLU 20-0262	MT232719	MT232714	—	—
*Distoseptispora tectonigena*	MFLUCC 12-0292 T	KX751714	KX751712	—	KX751709
*Distoseptispora terrestris*	HJAUP C2539 T	PV450538	PV448667	PV469764	
*Distoseptispora thailandica*	MFLUCC 16-0270 T	MH260292	MH275060	MH412767	—
*Distoseptispora thysanolaenae*	KUN-HKAS 112710	MW879524	MW723057	MW729783	—
*Distoseptispora thysanolaenae*	KUN-HKAS 102247 T	MK064091	MK045851	MK086031	—
*Distoseptispora tongrensis*	GMBC5343T	PV932989	PV932970	—	PX373360
*Distoseptispora tropica*	GZCC 22-0076 T	ON527943	ON527935	ON533679	ON533687
*Distoseptispora uncariicola*	UESTCC 24.0229T	PQ184739	PQ189784	PQ346521	PQ380002
*Distoseptispora vaginae*	GZCC 25-0527T	PV856180	PV820363	—	—
*Distoseptispora velvetica*	GZCC 24-0286T	PV856181	PV820364	—	—
*Distoseptispora verrucosa*	GZCC 20-0434 T	MZ868762	MZ868771	MZ892977	MZ892983
*Distoseptispora wuyishanensis*	HJAUP C2515 T	PV450537	PV448666	PV469763	PV469759
*Distoseptispora wuzhishanensis*	GZCC 22-0077 T	ON527946	ON527938	ON533682	—
*Distoseptispora xingpingensis*	KUNCC 22-12669	OQ732680	OQ874969	—	—
*Distoseptispora xingpingensis*	KUNCC 22-12667 T	OQ732681	OQ874970	OR367671	OQ870340
*Distoseptispora xishuangbannaensis*	KUMCC 17-0290 T	MH260293	MH275061	MH412768	MH412754
*Distoseptispora yichunensis*	HJAUP C1065 T	OQ942892	OQ942885	OQ944453	OQ944459
*Distoseptispora yongxiuensis*	JAUCC 4725 T	OK562394	OK562388	OK562406	—
*Distoseptispora yongxiuensis*	JAUCC 4726 T	OK562395	OK562389	OK562407	—
*Distoseptispora yunjushanensis*	JAUCC 4723 T	OK562398	OK562392	OK562410	—
*Distoseptispora yunjushanensis*	JAUCC 4724 T	OK562399	OK562393	OK562411	—
*Distoseptispora yunnansis*	MFLUCC 20-0153 T	MW081546	MW081541	MW084995	MW151861
*Distoseptispora zhejiangensis*	HJAUP C2588 T	PV450539	PV448668	PV469765	—
*Distoseptispora zunyiensis*	GZCC 23-0734T	—	PQ248941	PQ605053	PQ614176
*Fluminicola saprophytica*	MFLUCC 15-0976 T	MF374367	MF374358	MF370956	MF370954
*Myrmecridium banksiae*	CBS 132536 T	JX069855	JX069871	—	—
*Myrmecridium schulzeri*	CBS 100.54	EU041826	EU041769	—	—
*Papulosa amerospora*	AFTOL-ID 748	DQ470950	—	DQ471069	DQ470901
*Pleurophragmium bambusinum*	MFLUCC 12-0850	KU863149	KU940161	KU940213	—
*Pseudostanjehughesia aquitropica*	MFLUCC 16-0569 T	MF077559	MF077548	MF135655	—
*Pseudostanjehughesia aquitropica*	MFLUCC 15-0352 T	MK849787	MK828643	MN194047	MN124534
*Wongia griffinii*	DAR 80512 T	KU850471	KU850473	—	—

Notes: New species are in bold, and type strains are indicated with “T”. A dash (—) indicates data not available.

Maximum likelihood analysis was performed in the CIPRES Science Gateway v.3.3 (http://www.phylo.org/portal2/, Miller et al. 2010) using RAxML v. 8.2.8 as part of the “RAxML-HPC2 on XSEDE” tool (Stamatakis 2006, 2008). All free model parameters were estimated by RAxML using ML estimates of 25 per-site rate categories. The final ML was performed using the GTRGAMMA + I model.

Bayesian analysis was carried out with MrBayes v 3.1.2. (Ronquist et al. 2012) The best-fit model of evolution was determined using MrModeltest 2.2 (Nylander 2004). Posterior probabilities (PP) were calculated using Markov Chain Monte Carlo Sampling (MCMC) sampling in MrBayes v.3.1.2. Six simultaneous Markov chains were run for 10 million generations, with trees sampled every 100^th^ generation, resulting in 10,000 trees). The first 20,000 trees, representing the burn-in phase, were discarded, and the remaining 80,000 trees were used to calculate posterior probabilities in a majority-rule consensus tree.

Phylogenetic trees were visualized using FigTree v1.4.4 (Rambaut 2012), and figure layouts were prepared with Adobe Illustrator CS5 v.16.0.0 and Adobe Photoshop 2021 software (Adobe Systems, California, USA).

### Genealogical concordance phylogenetic species recognition (GCPSR) analysis

Genealogical concordance phylogenetic species recognition (GCPSR) analysis is a model used to detect significant recombinant events (Tian et al. 2024). The data (ITS-LSU-*rpb*2-*tef*1-α) were analyzed using SplitsTree V4 with the pairwise homoplasy index (PHI) test to estimate recombination levels among closely related species (Bruen et al. 2006). A multi-locus concatenated dataset including closely related species was used for the analysis. Relationships among taxa were visualized by constructing split graphs using the LogDet transformation and splits decomposition options. PHI values below 0.05 (Φw < 0.05) indicate significant recombination within the dataset.

## Results

### Phylogenetic analyses

**Figure 1.**
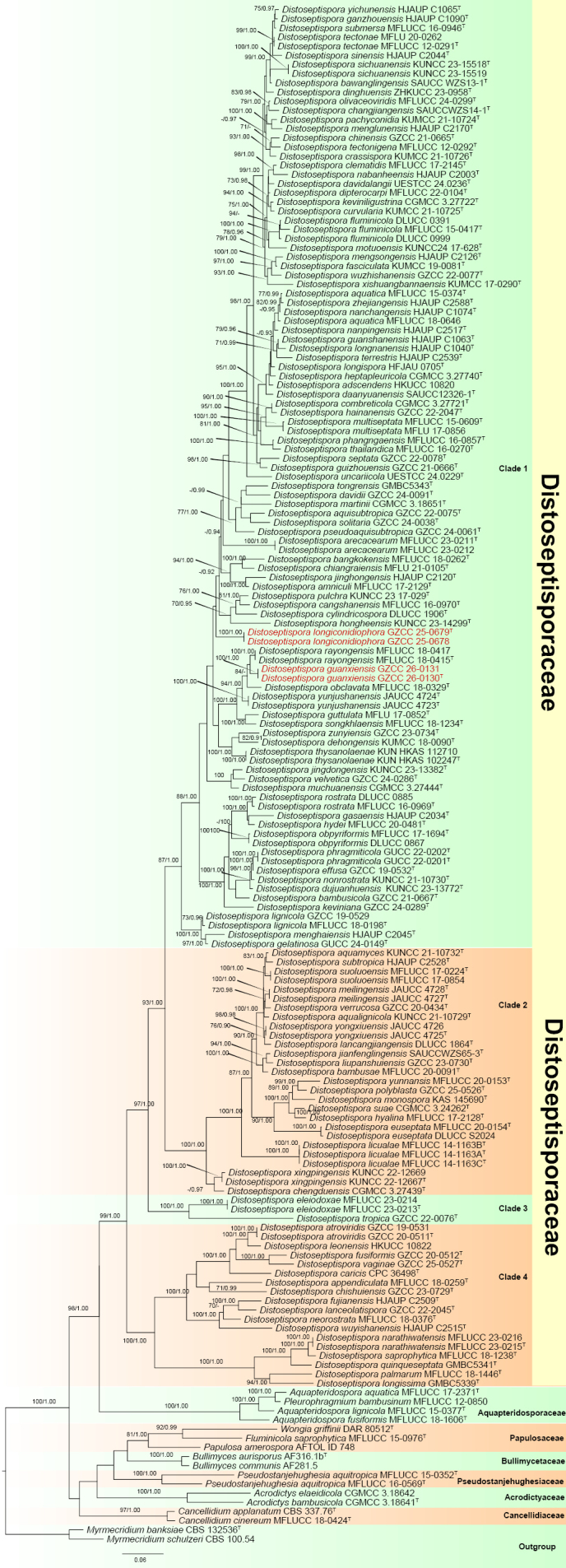
Maximum likelihood (ML) tree based on combined LSU, ITS, *rpb*2, and *tef*1-α sequence data. Bootstrap support values ≥ 70% and Bayesian posterior probabilities (PP) ≥ 0.90 are shown above the nodes as “ML/PP”. The tree is rooted with *Myrmecridium banksiae* (CBS 132536) and *M. schulzeri* (CBS 100.54). New species are in red, and type strains are indicated with “T”.

The combined ITS, LSU, *rpb*2, and *tef*1-α locus dataset comprised 166 taxa with *Myrmecridium banksiae* (CBS 132536) and *M. schulzeri* (CBS 100.54) designated as outgroup taxa. The RAxML analysis of the combined dataset yielded the best-scoring tree (Fig. 1) with a final ML optimization likelihood value of -55401.814741. The aligned dataset comprised 3450 characters, including gaps (ITS: 589 bp, LSU: 891 bp, *rpb*2: 1028 bp, *tef*1-α: 939 bp). The matrix contained 2009 distinct alignment patterns, with 27.69% of characters undetermined or missing. Estimated base frequencies were as follows: A = 0.239150, C = 0.266334, G = 0.280957, and T = 0.213559. Substitution rates were AC = 1.380627, AG = 3.693718, AT = 1.322136, CG = 0.915360, CT = 6.756680, and GT = 1.000000. The gamma distribution shape parameter (α) was 0.286845.

The Bayesian analysis yielded a tree with the same topology and clades as the ML tree. Nodes with maximum likelihood (ML) support ≥ 70% and Bayesian posterior probabilities (PP) ≥ 0.90 are indicated.

The multi-locus phylogenetic analysis showed that *Distoseptispora* species clustered into four distinct, well-supported clades (Fig. 1), consistent with recent studies (Shen et al. 2024). Our new isolates clustered within clade 1. *Distoseptispora longiconidiophora* (GZCC 25-0678 and GZCC 25-0679) and *D. guanxiensis* (GZCC 26-0130 and GZCC26-0131) were resolved in different lineages within *Distoseptispora*. *Distoseptispora longiconidiophora* (GZCC 25-0678 and GZCC 25-0679) grouped with *D. cylindricospora* (DLUCC 1906) and *D. hongheensis* (KUNCC 23-14299) with 30% ML statistical support (Fig. 1). *Distoseptispora guanxiensis* (GZCC 26-0130 and GZCC26-0131) clustered as a sister taxon to *D. rayongensis* (MFLUCC 18-0415 and MFLUCC 18-0417) with 52% ML and 0.71 PP statistical support (Fig. 1).

### Taxonomy

#### Distoseptispora guanxiensis

Taxon classificationFungiDistoseptisporalesDistoseptisporaceae

D.F. Bao & X.G. Tian sp. nov.

F982A53E-85DF-5656-BFD1-34AB2CF86D24

Index Fungorum: IF905148

Fig. 2

##### Etymology.

Refers to the type location, Guangxi Zhuang Autonomous Region, China.

**Figure 2.**
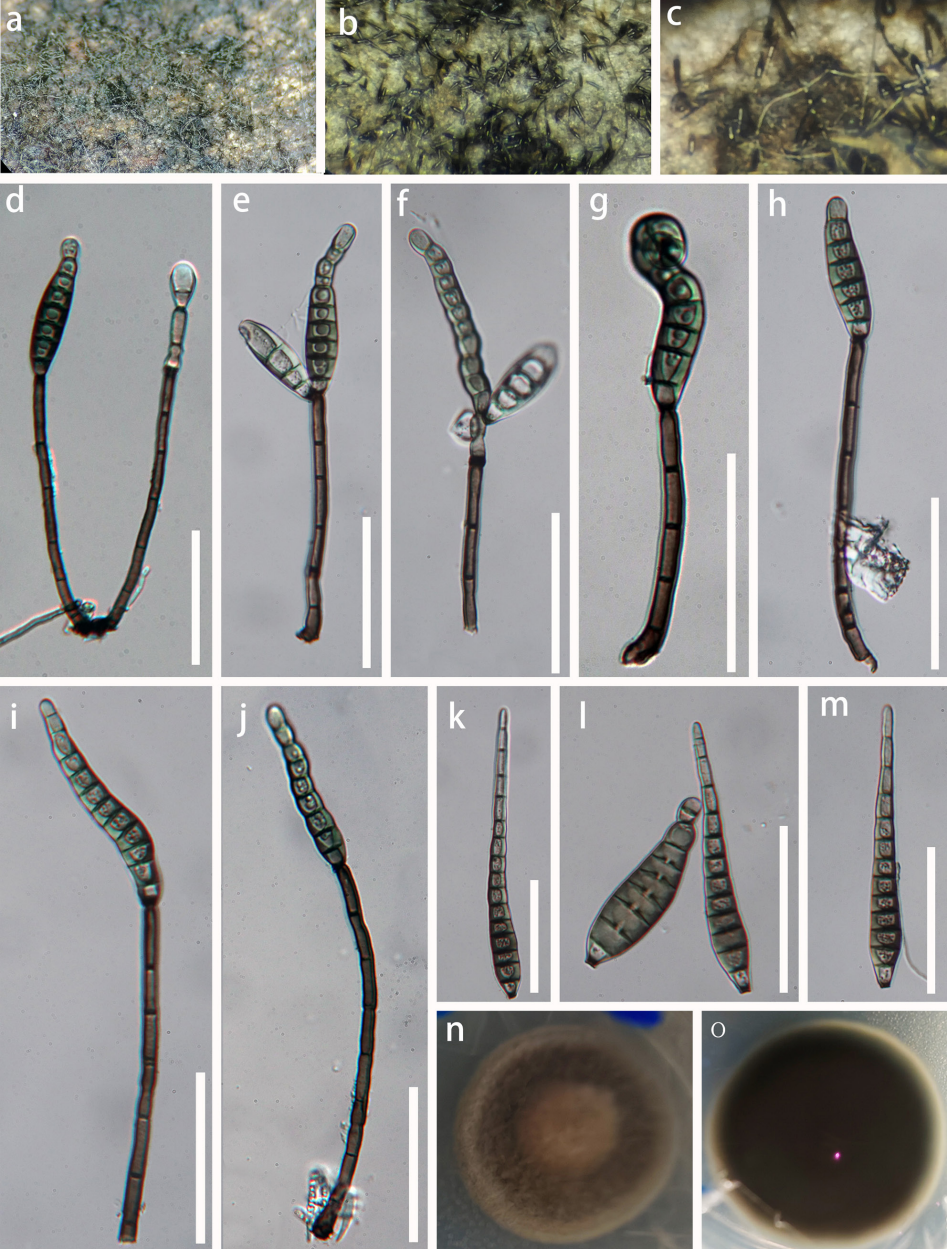
*Distoseptispora guanxiensis* (GZAAS 25-0710, holotype). **a–c**. Colonies on substrate; **d–g, i**. Conidia attached to conidiophores; **h**. A conidiophore; **j–l**. Conidia; **m**. Surface view of colonies on PDA; **n**. Reverse view of colonies on PDA. Scale bars: 50 μm (d, m).

##### Holotype.

GZAA 25-0710.

##### Description.

***Saprobic*** on decayed, submerged wood in freshwater habitats. **Sexual morph**: Undetermined. **Asexual morph**: Hyphomycetous. ***Colonies*** effuse, olivaceous or dark brown, scattered, hairy. ***Mycelium*** semi-immersed, composed of septate, smooth, branched, subhyaline to pale brown hyphae. ***Conidiophores*** 79.5–141 × 3.2–5.5 µm (= 107 × 4.3 μm, n = 10), macronematous, mononematous, erect, solitary, cylindrical, rounded at the apex, slightly swollen at the base, straight or slightly flexuous, septate, olivaceous or brown, smooth-walled. ***Conidiogenous cells*** 11–21.7 × 3.7–5.5 µm (= 17.6 × 4.5 μm, n = 10), holoblastic, monoblastic, integrated, determinate, terminal, cylindrical, brown. ***Conidia*** 36–122 × 7.5–15 µm (= 70 × 11.4 μm, n = 20), acrogenous, solitary, obclavate or obspathulate, rostrate, tapering towards the apex, straight or slightly curved, olivaceous to brown, becoming pale brown to subhyaline towards the apex, truncate at the base, 7–15-distoseptate, smooth-walled, guttulate, sometimes with percurrent proliferation forming a secondary conidium from the conidial apex.

##### Culture characteristics.

Colonies on PDA reaching 3.5 cm diam. after 4 weeks at room temperature, grayish brown to brown from above, circular, smooth, velvety; reverse black, brown to dark brown from below.

##### Material examined.

China • Guangxi Zhuang Autonomous Region, Fangchenggang City, Shangsi County, on decaying wood submerged in a River, 3 Oct. 2025, D.F. Bao, B580 (GZAAS 26-0001, holotype), ex-type, GZCC26-0130; • ibid, B581 (GZAAS 26-00012, isotype), ex-isotype, GZCC26-0131.

##### Notes.

**Figure 3.**
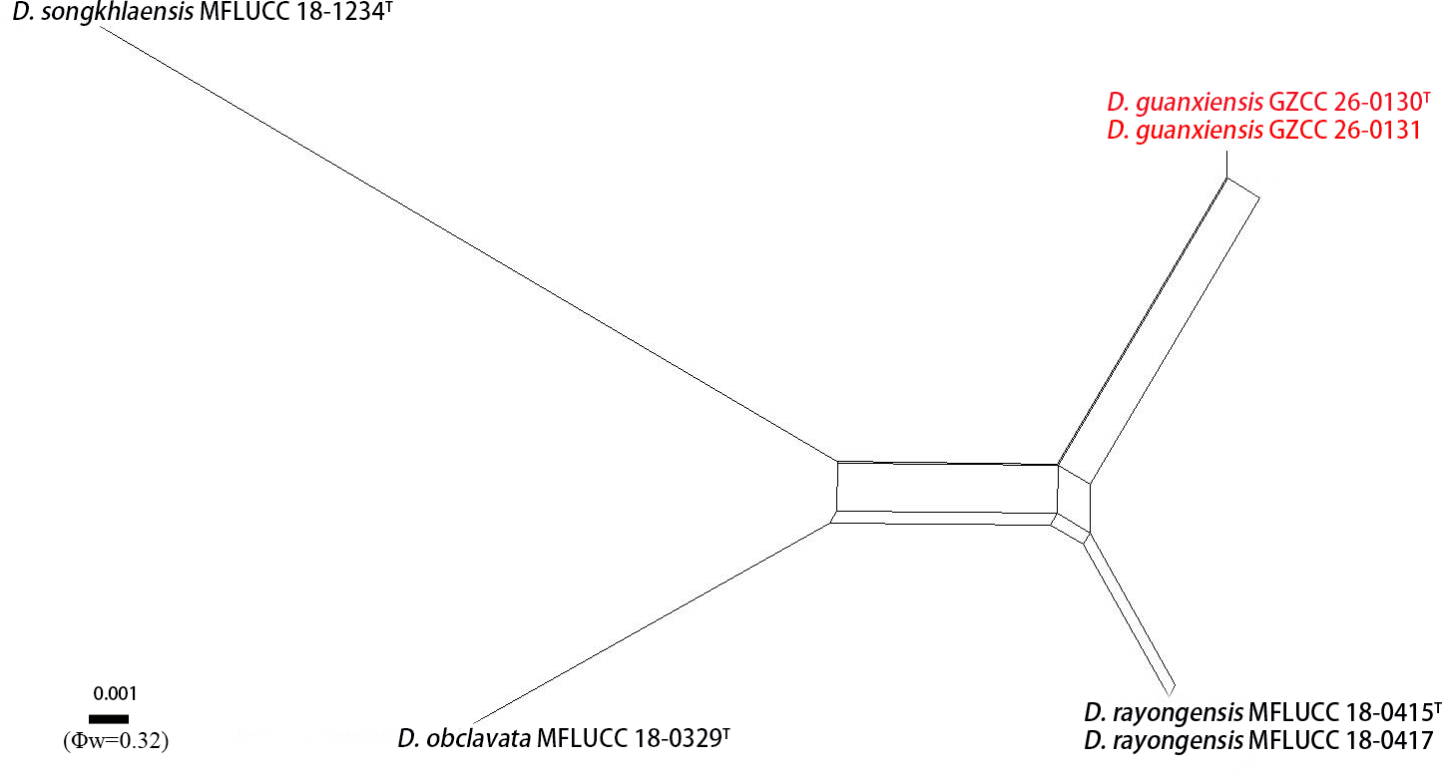
Results of the PHI test of *Distoseptispora guanxiensis* and closely related species using both LogDet transformation and splits decomposition. New strains are in red, and “^T^” indicates holotype or ex-type strains.

In our phylogenetic analyses, *Distoseptispora guanxiensis* (GZCC 26-0130 and GZCC 26-0131) clustered sister to *D. rayongensis* (MFLUCC 18-0417 and MFLUCC 18-0415) with 30% ML statistical support. *Distoseptispora guanxiensis* is morphologically similar to *D. rayongensis* in having macronematous, mononematous, cylindrical, smooth-walled, septate, unbranched, pale to dark brown conidiophores, monoblastic, integrated, terminal, determinate conidiogenous cells and acrogenous, solitary, septate, obclavate or obspathulate conidia (MFLU 18-1045, holotype). However, the conidiogenous cells of our new isolate are polyblastic, while they are monoblastic in *D. rayongensis*. In addition, the conidiophores (79.5–141 vs. 75–125 μm) and conidia (36–122 vs. 60–106 μm) of *D. guanxiensis* are longer than those of *D. rayongensis* (Hyde et al. 2020). The comparisons of ITS, LSU, *rpb*2 and *tef*1-α sequence data between our new isolate and *D. rayongensis* (MFLUCC 18-0417), revealed 16 bp (2.9%), 8 bp (0.97%), 24 bp (2.3%), and 6 bp (0.67%) differences without gaps, respectively. The PHI test (Φw = 0.32) indicated no significant recombination events between our strains (GZCC 25-0679 and GZCC 25-0678) and their closely related taxa (Fig. 3). Both *D. guanxiensis* and *D. rayongensis* were collected from freshwater habitats, however, *D. guanxiensis* was collected from China while, *D. rayongensis* was from Thailand. Based on morphological differences, phylogenetic analysis and PHI analysis, we introduce *D. guanxiensis* as a new species.

#### Distoseptispora longiconidiophora

Taxon classificationFungiDistoseptisporalesDistoseptisporaceae

D.F. Bao & X.G. Tian sp. nov.

32C74CC7-2F7A-5A85-9DF1-10D47406098E

Index Fungorum: IF905074

Fig. 4

##### Etymology.

Refers to the long conidiophores of the new species.

**Figure 4.**
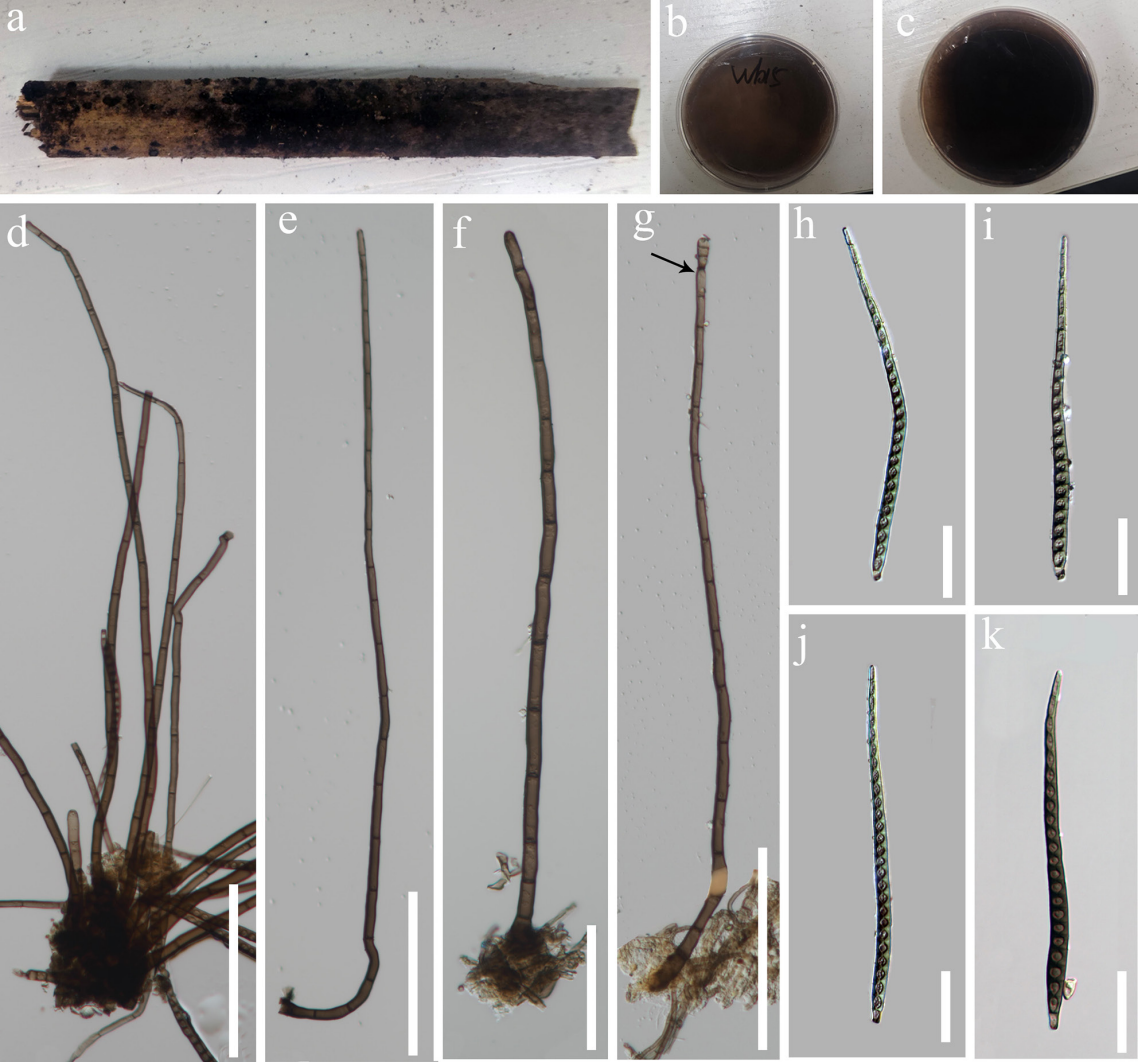
*Distoseptispora longiconidiophora* (GZAAS 25-0709, holotype) **a**. Colonies on substrate; **b**. Colonies on PDA (front view); **c**. Colonies on PDA (reverse view); **d–f**. Conidiophores; **g**. Conidium attached to conidiophore; **h–k**. Conidia; Scale bars: 50 μm (d–g); 30 μm (h–k).

##### Holotype.

GZAAS 25–0709.

##### Description.

***Saprobic*** on decaying, submerged wood. **Sexual morph**: Undetermined. **Asexual morph: *Colonies*** on the substratum superficial, gregarious, effuse, scattered, hairy, brown to dark brown. ***Mycelium*** mostly immersed, composed of branched, septate, brown to dark brown, smooth hyphae. ***Conidiophores*** 196–409 (–486) × 4.5–6.5 μm (= 302.6 × 5.6 μm, n = 25), macronematous, mononematous, erect, cylindrical, solitary or in a small groups, straight or slightly flexuous, unbranched, 7–21-septate, smooth, dark brown. ***Conidiogenous cells*** 9.5–25.5 (–48) × 3.5–5 μm (= 17.5 × 4.3 μm, n = 25), holoblastic, monoblastic, integrated, determinate, terminal, cylindrical, brown to dark brown. ***Conidia*** 100–142 × 5.5–8 μm (= 120.8 × 7.0 μm, n = 25), acrogenous, solitary, dry, cylindrical to obclavate, straight or slightly curved, truncate at the base, tapering towards the apex, 13–27-distoseptate, olivaceous-brown to dark brown, smooth, thick and smooth-walled, without a mucilaginous sheath.

##### Culture characteristics.

Colonies on PDA reaching 3.5 cm diam. after 4 weeks at room temperature, brown to dark brown from above, circular, smooth; reverse black, dark brown to black from below.

##### Material examined.

China • Yunnan Province, Xishuangbanna City, on decaying submerged wood, 17 Sep. 2021, X.G. Tian, wb15 (GZAAS 25-0709, holotype), ex-type, GZCC 25-0679; • ibid, 20 Sep. 2021, X.G. Tian, wb20 (GZAAS 25-0708, paratype), ex-paratype, GZCC 25-0678.

##### Notes.

In our phylogenetic analyses, the new isolates, *Distoseptispora longiconidiophora* (GZCC 25-0679 and GZCC 25-0678) formed a distinct lineage within the clade 1, and close to *D. cangshanensis*, *D. cylindricospora*, *D. hongheensis* and *D. pulchra* (Fig. 1). However, *Distoseptispora longiconidiophora* can be easily distinguished from the *D. cangshanensis* and *D. pulchra* by its conidia (100–142 vs. 196–409 μm) are shorter than conidiophores. In contrast, conidia of *D. cangshanensis* and *D. pulchra* are longer than their conidiophores (Luo et al. 2018; Ma et al. 2022). In addition, *D. longiconidiophora* has longer conidiophores (196–409 [–486] vs. 44-68 μm) with more septa (7–21 vs. 1–5) and narrower conidia (5.5–8 vs. 10–14 μm) than those of *D. cangshanensis* (Luo et al. 2018). Similarly, *D. longiconidiophora* has longer conidiophores (196–409 [–486] vs. 47–62 μm) and more conidial septa (13–27 vs. 3–14) than *D. pulchra*.

**Figure 5.**
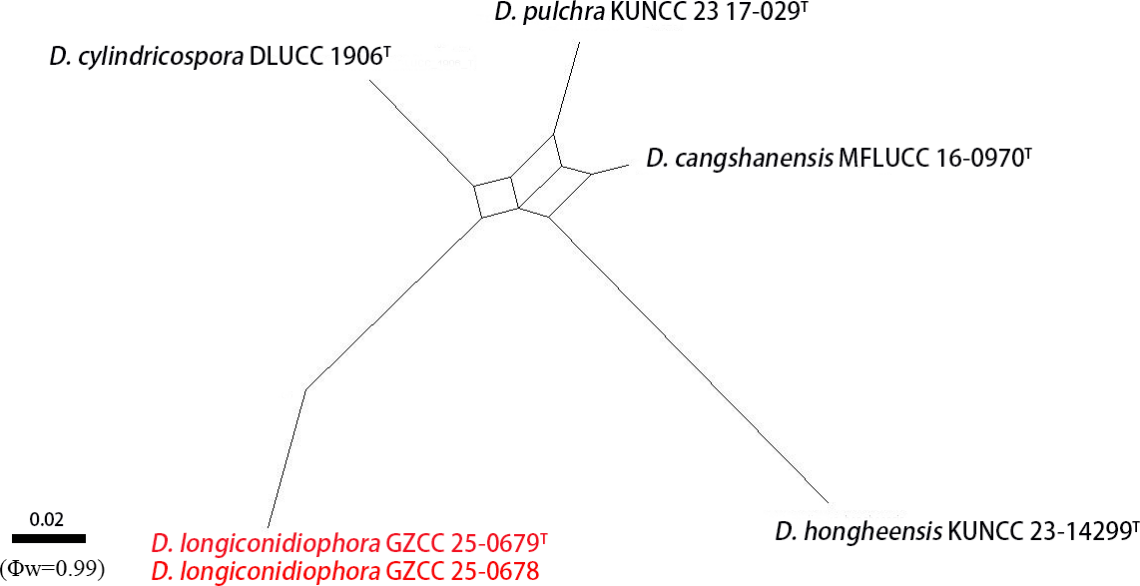
Results of the PHI test of *Distoseptispora longiconidiophora* and closely related species using both LogDet transformation and splits decomposition. New strains are in red, and “^T^” indicates holotype or ex-type strains.

Conidia of *Distoseptispora longiconidiophora*, *D. cylindricospora*, and *D. hongheensis* are all shorter than their conidiophores. However, *D. longiconidiophora* differs from *D. cylindricospora* in having much longer conidiophores (196–409 [–486] vs. 105–157 μm) with fewer septa (7–21 vs. 18–30), and fewer conidial septa (13–27 vs. 20–65). *Distoseptispora longiconidiophora* can be distinguished from *D. hongheensis* in having longer conidiophores with more septa and narrower conidia (5.5–8 vs. 8–10 μm). Additionally, the PHI test (Φw = 0.99) also indicates no significant recombination events between our strains (GZCC 25-0679 and GZCC 25-0678) and their closely related taxa (Fig. 5). Both phylogenetic analysis, morphological evidence, and PHI analysis support our new isolates as representing a new species.

## Discussion

This study provides an updated taxonomic and phylogenetic framework for *Distoseptispora* by introducing two novel species, *D. guanxiensis* and *D. longiconidiophora*, isolated from submerged wood in southern China. The classification of these species is robustly supported by integrative morphological and molecular evidence. Among the 124 known species of *Distoseptispora*, 73 records have been reported from China, particularly the provinces of Guizhou and Yunnan.

Zhang et al. (2022) demonstrated that *Distoseptispora* clustered into three well-supported clades via phylogenetic analyses, correlating morphological traits such as conidiophore length and proliferation, conidial septation type (distoseptate or euseptate), conidial shape and size, and ecological preference. However, they were unable to identify consistent morphological markers that reliably delineated the clades. Our study extends this framework, revealing four clades (Fig. 1), consistent with recent findings (Shen et al. 2024; Dong et al. 2025). Notably, the newly defined Clade 3 comprises two species, *D. eleiodoxae* (MFLUCC 23-0214) and *D. tropica* (GZCC 22-0076), both exhibit obpyriform, rostrate conidia (Ma et al. 2022; Karimi et al. 2024), which are distinct from those in other clades. Morphologies of Clades 1 and 2 overlap, with both producing thick-walled, obclavate conidia with variable shapes. For example, conidia of *D. hydei* (MFLUCC 20–0115) are obpyriform and fusiform; *D. obpyriformis* (MFLUCC 17-1694) produces obpyriform conidia; whereas conidia of *D. martinii* (CGMCC 3.18651) are ellipsoid, oblate or subglobose. Clade 4 primarily has thin-walled, euseptate, obclavate conidia, only *D. meilingensis* (JAUCC 4727) exhibit thick-walled conidia (Zhai et al. 2022). Conidiogenous cell proliferation occurs across all clades, further complicating classification. In addition, species in Clade 1 produce long conidia (> 100 μm), whereas Clade 4 species bear shorter conidia (< 100 μm). The inconsistent distribution of traits, such as septation and wall thickness, suggests evolutionary mechanisms, including convergent evolution, that warrant further investigation through integrative genomic and ecological studies.

Our newly described species, *D. longiconidiophora* and *D. guanxiensis*, clustered within Clade 1 (Fig. 1). This placement aligns with the morphological trends documented by Zhang et al. (2022), where Clade 1 species typically produce elongated conidia (mostly > 100 μm, occasionally reaching up to 700 μm) with numerous septa (often exceeding 20, up to 80). The combined phylogenetic and morphological evidence underscores the importance of integrative approaches that reconcile molecular data with detailed morphological examination. Future research should prioritize expanded biogeographic sampling, resolution of cryptic speciation using genomic tools. This work not only refines the taxonomic boundaries of the genus but also reinforces the value of integrating phylogenomics with morphological examination to advance fungal systematics and conservation biology.

## Supplementary Material

XML Treatment for Distoseptispora guanxiensis

XML Treatment for Distoseptispora longiconidiophora

## Additional information

### Artificial Intelligence (AI) use

The authors accept full responsibility for the content of the manuscript, including the disclosure of any use of AI.

No AI tools were used in the preparation of this manuscript.

### Author ORCIDs

X.-G. Tian https://orcid.org/0000-0002-8124-1281

J.-J. Han https://orcid.org/0009-0000-4919-369X

Y.-Z. Lu https://orcid.org/0000-0002-1033-5782

S.C. Karunarathna https://orcid.org/0000-0001-7080-0781

S. Tibpromma https://orcid.org/0000-0002-4706-6547

D.-F. Bao https://orcid.org/0000-0002-5697-4280

### Data availability

All of the data that support the findings of this study are available in the main text.
